# Family‐based treatment of children with severe obesity in a public healthcare setting: Results from a randomized controlled trial

**DOI:** 10.1111/cob.12513

**Published:** 2022-02-25

**Authors:** Hanna F. Skjåkødegård, Rachel P. K. Conlon, Sigurd W. Hystad, Mathieu Roelants, Sven J. G. Olsson, Bente Frisk, Denise E. Wilfley, Yngvild S. Danielsen, Petur B. Juliusson

**Affiliations:** ^1^ Department of Clinical Science University of Bergen Bergen Norway; ^2^ Department of Psychiatry University of Pittsburgh School of Medicine Pittsburgh Pennsylvania USA; ^3^ Department of Psychosocial Science University of Bergen Bergen Norway; ^4^ Department of Public Health and Primary Care, KU Leuven University of Leuven Leuven Belgium; ^5^ Independent Researcher Stockholm Sweden; ^6^ Department of Health and Functioning Western Norway University of Applied Sciences Bergen Norway; ^7^ Department of Physiotherapy Haukeland University Hospital Bergen Norway; ^8^ Department of Psychiatry Washington University School of Medicine St. Louis Missouri USA; ^9^ Department of Clinical Psychology University of Bergen Bergen Norway; ^10^ Children and Youth Clinic Haukeland University Hospital Bergen Norway; ^11^ Department of Health Registry Research and Development Norwegian Institute of Public Health Bergen Norway

**Keywords:** adolescent, behavioural treatment, children, family‐based treatment, paediatric obesity, randomized controlled trial

## Abstract

To compare the effectiveness of family‐based behavioural social facilitation treatment (FBSFT) versus treatment as usual (TAU) in children with severe obesity. Parallel‐design, nonblinded, randomized controlled trial conducted at a Norwegian obesity outpatient clinic. Children aged 6–18 years referred to the clinic between 2014 and 2018 were invited to participate. Participants were randomly allocated using sequentially numbered, opaqued, sealed envelopes. FBSFT (*n* = 59) entailed 17 sessions of structured cognitive behavioural treatment, TAU (*n* = 55) entailed standard lifestyle counselling sessions every third month for 1 year. Primary outcomes included changes in body mass index standard deviation score (BMI SDS) and percentage above the International Obesity Task Force cut‐off for overweight (%IOTF‐25). Secondary outcomes included changes in sleep, physical activity, and eating behaviour. From pre‐ to posttreatment there was a statistically significant difference in change in both BMI SDS (0.19 units, 95% confidence interval [CI]: 0.10–0.28, *p* < .001) and %IOTF‐25 (5.48%, 95%CI: 2.74–8.22, *p* < .001) between FBSFT and TAU groups. FBSFT participants achieved significant reductions in mean BMI SDS (0.16 units, (95%CI: −0.22 to −0.10, *p* < .001) and %IOTF‐25 (6.53%, 95% CI: −8.45 to −4.60, *p* < .001), whereas in TAU nonsignificant changes were observed in BMI SDS (0.03 units, 95% CI: −0.03 to 0.09, *p* = .30) and %IOTF‐25 (−1.04%, 95% CI: −2.99 to −0.90, *p* = .29). More FBSFT participants (31.5%) had clinically meaningful BMI SDS reductions of ≥0.25 from pre‐ to posttreatment than in TAU (13.0%, *p* = .021). Regarding secondary outcomes, only changes in sleep timing differed significantly between groups. FBSFT improved weight‐related outcomes compared to TAU.

**What is already known about this subject**

- Family‐based behavioural treatment is recommended as an evidence‐based treatment for childhood obesity.
- Family‐based behavioural treatment delivered in research clinics, has been shown to yield clinically significant weight loss in children with obesity.

**What this study adds**

- Delivered at an obesity outpatient clinic, family‐based behavioural social facilitation treatment (FBSFT) improved weight‐related outcomes significantly more than treatment as usual (TAU) among children (ages 6–18 years) with severe obesity.
- Investigation of individual treatment response showed that significantly more children receiving FBSFT achieved a clinically meaningful body mass index standard deviation score reduction of ≥0.25 compared to children receiving TAU.
- The beneficial changes in weight outcomes exhibited in FBSFT compared to TAU were not explained by differences in sleep, physical activity, or eating behaviour.

## 1 INTRODUCTION

Paediatric obesity is one of the major global health challenges of the 21st century. Effective treatment options are urgently needed, given the increasing prevalence of paediatric obesity, the risk of adverse health consequences, and the fact that obesity‐related risk factors track into adulthood.^1^, ^2^

To date, various treatment options have been tested, including interventions focusing on lifestyle modification, as well as pharmacological therapy and bariatric surgery,^1^, ^3^, ^4^ which are summarized in numerous reviews and meta‐analyses.^3^, ^4^, ^5^, ^6^, ^7^, ^8^ Despite this rapidly growing body of treatment research, studies have yielded similar findings over the last decades,^1^, ^9^ and lifestyle modification has remained the preferred treatment strategy for children and adolescents.^1^, ^10^, ^11^

Treatment programmes targeting multiple lifestyle behaviours whilst applying behavioural techniques in a family‐based approach have shown the most promise and are considered best practise in obesity treatment for children aged 6–17 years.^10^, ^12^, ^13^ However, evidence is mostly derived from efficacy trials in research clinics, with strict control of internal validity and participant selection.^14^, ^15^ An important next step is to conduct effectiveness trials focusing on treatment delivery in public healthcare settings with less stringent participant selection criteria.^9^, ^11^ Evidence for the effectiveness of such treatment programmes that can be extrapolated to different healthcare settings is sparse,^16^, ^17^ and sought by national health authorities.^18^ The family‐based behavioural treatment of childhood obesity (FABO) study aimed to address this need. The study enrolled children who met the criteria for admission to a tertiary care obesity clinic within the public healthcare service in Norway,^19^ and compared family‐based behavioural social facilitation treatment (FBSFT) with treatment as usual (TAU) (comprising lifestyle intervention, including diet and physical activity). This study design provided the opportunity to investigate the FBSFT approach in a growing, but often overlooked, patient population of children with the most severe form of obesity^20^ (International Obesity Task Force [IOTF] body mass index [BMI] ≥35 or ≥30^21^ with comorbidity).

Moreover, emerging data suggest that obesity risk is influenced by sleep patterns. Several aspects of sleep, including duration and timing, have been identified as contributors to the development and maintenance of childhood obesity.^22^ However, family‐based lifestyle interventions usually target diet and physical activity, and less commonly sleep.^13^, ^23^ A recent review found that only 20% of 119 family‐based intervention studies included a sleep component, usually in children aged 2–5 years,^23^ with most studies assessing sleep using parent‐reported data. As sleep problems may impact the effectiveness of treatment interventions, evaluating sleep patterns, along with changes in other lifestyle behaviours (e.g., eating behaviour, physical activity), will inform our understanding of key treatment components that are critical to target in family‐based obesity interventions.

The aim of the present study was to compare the effectiveness of FBSFT to TAU in severe childhood obesity treatment delivered in a public healthcare setting. Outcome measures included BMI‐related metrics, sleep measures, physical activity, and eating behaviour. We hypothesized that FBSFT would yield greater improvements in BMI‐related metrics as well as sleep and eating behaviour, compared to TAU, with similar improvements in physical activity due to a comparable focus on this component in both treatment programmes.

## 2 METHODS

### 2.1 Study design

**FIGURE 1:**
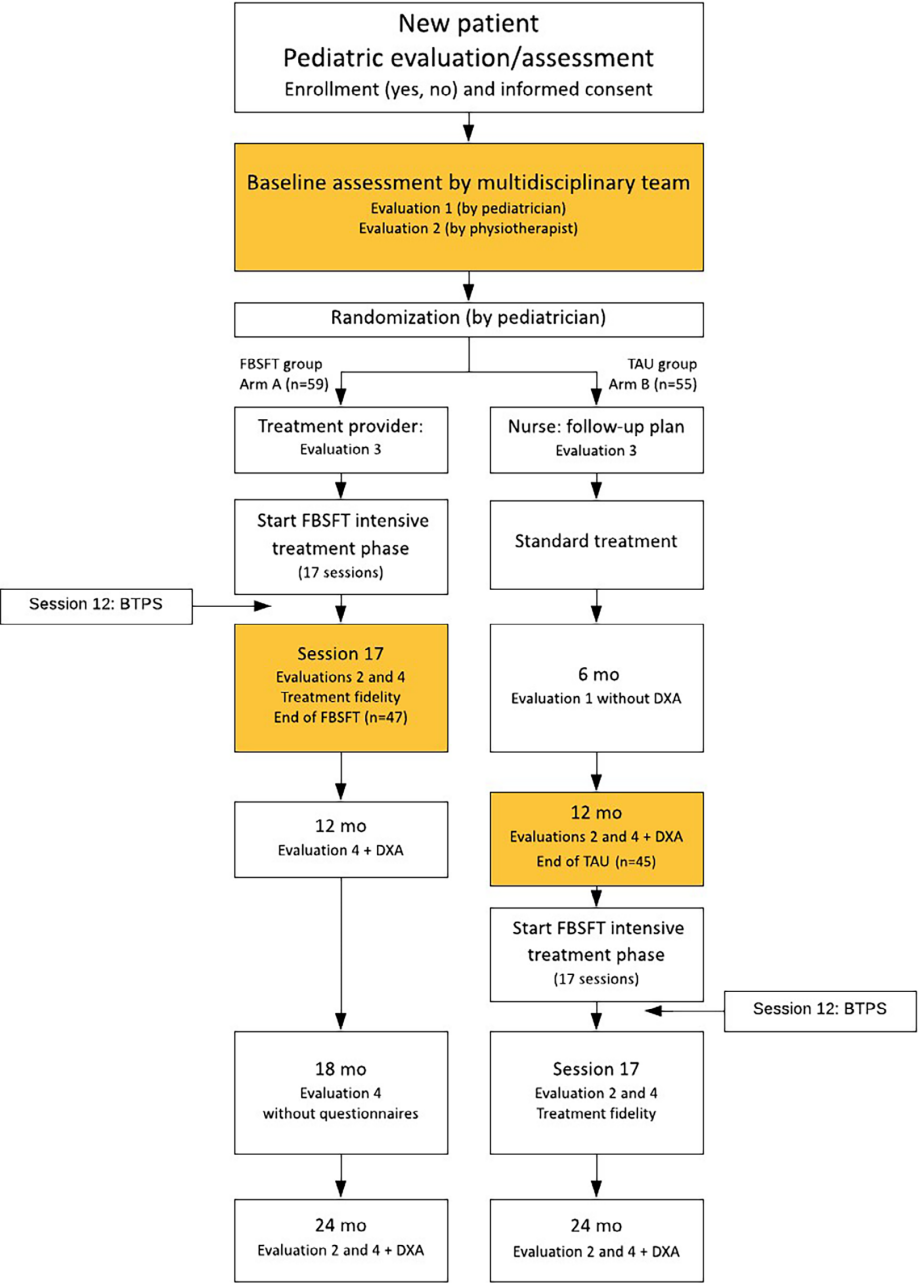
Flow chart showing the FABO study design (modified from study protocol previously described).^19^ Coloured boxes represent study measurement time points. Evaluation 1: DXA, BIA, BP, height, weight, waistC. Evaluation 2: actigraphy, sleep and physical activity. Evaluation 3: questionnaire assessment (DEBQ, YEDE‐Q, YSR, CBCL, CDI, SPPC). Evaluation 4: BIA, BP, height, weight, waistC, questionnaire assessment (as for Evaluation 3). BTPS: applied after 12 FBSFT sessions and in dropout population. Abbreviations: BIA, bioelectrical impedance analysis; BP, blood pressure; BTPS, Barriers to Treatment Participation Scale; CBCL, Child Behaviour Checklist; CDI, Children's Depression Inventory; DEBQ, Dutch Eating Behaviour Questionnaire; DXA, dual‐energy X‐ray absorptiometry; FBSFT, family‐based behavioural social facilitation treatment; mo, months; SPPC, Self‐Perception Profile for Children; TAU, treatment as usual; waistC, waist circumference; YEDE‐Q, Youth Eating Disorder Examination Questionnaire; YSR, The Youth Self‐Report

The FABO study is a parallel‐design, nonblinded, randomized controlled trial (RCT), conducted at the Obesity Outpatient Clinic at Haukeland University Hospital, Bergen, Norway. All children (aged 6–18 years) referred by their general practitioner to the clinic between February 2014 and October 2018 were invited to participate. Written informed consent was obtained after an initial clinic assessment. Participating families were randomized to either FBSFT (Arm A) or TAU (Arm B). Randomization was in 1:1 ratio, and sequentially numbered, opaque sealed envelopes were used to conceal the randomization sequence. Figure 1 depicts the study design, including the primary measurement time points at baseline and after FBSFT (Arm A) or 1 year of TAU (Arm B).

The study was approved by the Regional Committee for Medical and Health Research Ethics, Western Norway (number 2013/1300) and was registered on ClinicalTrials.gov (NCT02687516).

### 2.2 Participants

One hundred fourteen children and adolescents (mean age 12.6 years; minimum–maximum: 5.9–17.7 years) participated, with 59 participants in Arm A and 55 in Arm B. Inclusion criteria were BMI above the IOTF cut‐off for severe obesity (≥35 kg/m^2^) or for obesity (≥30 kg/m^2^)^21^ in the presence of weight‐related comorbidities. The family‐based approach to this intervention required that both the child and at least one parent agreed to actively participate. Parental weight status was not assessed prior to inclusion. Families were excluded if either the child or one or both parents experienced severe somatic or psychiatric illness affecting weight or adherence to the treatment programme, or if the child was participating in other obesity treatment programmes.^19^

### 2.3 Description of treatments

FBSFT focuses on promoting healthy lifestyle behaviours and attitudes using a combination of behavioural and cognitive techniques. It builds on family‐based behavioural treatment (FBT) for paediatric obesity, which is the most documented approach in childhood obesity treatment^10^ and has been shown to yield clinically significant weight loss in children with obesity.^17^ FBSFT not only incorporates all of the features of FBT, which focuses on the individual, as well as on the family/home environment,^10^ but also extends the focus across socioecological contexts, thus supporting and sustaining changes in health behaviours.^19^ This extension includes evaluation and engagement of supports across the peer network and community levels such as school settings. FBSFT also includes elements of interpersonal therapy for eating disorders aimed at tackling emotions and interpersonal conflicts that affect eating habits. Health behaviours in terms of diet, physical activity, sedentary behaviour, and sleep are targeted in both children and their parents applying the Traffic Light Eating Plan and activity programme.^15^ Pretreatment measures of the mentioned lifestyle behaviours were used to form the basis for the planning of healthy changes. Progress was monitored from session‐to‐session using specific weight goals and relevant lifestyle behaviours adjusted thereafter. The goal for children aged ≤10 years was stable weight maintenance throughout the programme, whereas for children aged ≥10 years session‐to‐session weight reduction of 250 g was used as a reference point.

Traditionally, FBT is implemented in a mixed (group + individual) format,^15^ whilst FBSFT was delivered in 17 fortnightly individual family sessions (mean treatment duration 178 ± 47 days). Families had to attend 15 of 17 sessions to be considered completers. The majority of children were accompanied by one parent to each session. Parental participation was considered important for children of all ages, but individual adjustments related to age were implemented, providing adolescents with greater responsibility for healthy changes compared to younger participants. Families met with the same healthcare worker from the multidisciplinary team at the obesity clinic for all sessions. The team consisted of a paediatrician, nutritionist, physiotherapist, nurse and psychologist, and all team members were trained in FBSFT prior to treatment delivery. The intensive treatment phase, including session‐specific topics and application of behavioural and cognitive techniques, was delivered as previously described in the study protocol.^19^

Families assigned to TAU (Arm B), a lifestyle intervention targeting the child, were provided with a personalized plan for changing specific lifestyle behaviours and were advised to participate in monthly counselling sessions with their local healthcare nurse. TAU was delivered over the course of 12 months^19^ (mean treatment duration 374 ± 41 days) and included quarterly assessments, progress evaluation, and goal revision in clinic. Of the participants, 87% attended all the appointed assessments at the obesity clinic.

### 2.4 Anthropometric measures

Height and weight were measured by trained assessors in clinic. The assessors were informed about study participation, but not treatment assignment. Height was measured to the nearest 0.1 cm with an electronic wall‐mounted seca 264 stadiometer (Seca), and weight was measured to the nearest 0.1 kg using a digital InBody720 scale (Biospace). Measurements were taken with participants wearing light indoor clothing only (without socks and shoes).^19^ Weight status was assessed using two metrics converted from the BMI (kg/m^2^): BMI standard deviation score (SDS) and percentage above the IOTF cut‐off for overweight^21^ (%IOTF‐25). The BMI SDS was calculated using the Norwegian growth reference,^24^ whereas %IOTF‐25 is the percentage above the IOTF threshold for overweight based on a child's age and sex, calculated as 100 × (BMI/IOTF‐25).^25^ A cut‐off point of ≤−0.25 BMI SDS was used to define a clinically relevant change from pre‐ to posttreatment in participants from each group.^26^, ^27^

### 2.5 Sleep measures

Sleep was objectively measured using an Actiwatch 2 (Philips Respironics). Actiwatch 2 devices are wrist‐worn accelerometers with a light sensor and an event marker, which record all uniaxial movement over 0.05G.^28^ Data were collected using 30‐s epochs, and a medium sensitivity threshold was used to score the epochs as either “wake” or “sleep.” Medium sensitivity thresholds have been shown to yield the least biased estimates of wakefulness, total sleep time, and wake after sleep onset in school‐aged children.^28^ The device was worn on the wrist of the nondominant arm for 7 consecutive days pre‐ and posttreatment in both groups. Participants were instructed to press the event marker when switching off the light at night and on waking up in the morning. Actiwatch 2 has been validated, both in clinical sleep laboratories and in the natural home environment and is commonly used in sleep research in children aged 3–18 years.^29^, ^30^

Sleep statistics were calculated using Respironics Actiware software, version 6.0.9. The rest interval (time in bed) associated with the main sleep period in the 24‐h day was manually set, according to a standardized scoring protocol.^28^ Furthermore, sleep time within this interval was automatically detected by a standard default algorithm in the proprietary software.

#### 2.5.1 Sleep duration

The mean sleep duration over 7 consecutive days was used in the analyses. For inclusion, participants completed recordings of at least 5 (out of 7) days, including at least three school nights and two weekend nights. At baseline 105 of 114 participant presented with valid sleep recordings, 96 with 7 nights, 7 with 6 nights and 2 with 5 nights. Posttreatment the numbers where 79 of 114 participants in total, 66 with 7 nights, 11 with 6 nights and 2 with 5 nights.

#### 2.5.2 Sleep timing

Sleep timing (i.e., when sleep occurs) was calculated as a 7‐day mean of the mid‐sleep time, i.e., the midpoint between sleep onset time and wake‐up time: (sleep onset time + sleep offset time)/2. For participants with only five or six nights of recordings, the mean of these nights was used. Furthermore, sleep onset and final wake‐up times were reported for additional information about sleep timing. Seen together, these parameters give valuable information about sleep hygiene. Sleep during daytime was not assessed in the study.

### 2.6 Physical activity measures

Daytime physical activity (between 8:00 am and 9:00 pm) was objectively assessed using data from the same device (Actiwatch 2). Wrist‐worn accelerometers have been validated for measuring physical activity in children,^31^ and their use shown to maximize compliance.^32^

Data were downloaded using Respironics Actiware software, version 6.0.9, and exported into Microsoft Excel 2016 for further processing using a tailor‐made software. The collected activity data were categorized into different intensity levels based on previously used and validated cut‐off values: light (160–523 counts/30‐s epochs), moderate (524–811 counts/30‐s epochs), and vigorous intensity (>812/30‐s epochs).^33^ To be included in the analysis, participants needed ≥10 h of wear time each day and ≥4 days of recorded data.^33^ Physical activity level was calculated as the percentage of time spent on moderate‐to‐vigorous physical activity (MVPA).

### 2.7 Eating behaviour

#### 2.7.1 Dutch Eating Behaviour Questionnaire

The Dutch Eating Behaviour Questionnaire (DEBQ) is a measure of disturbed eating patterns in children and adolescents.^34^ Two versions of the questionnaire were used. An age‐adapted 20‐item version^35^ for children aged <10 years and a full 33‐item questionnaire for children aged ≥10 years.^34^ Both versions comprised three subscales: restrained, external, and emotional eating.^34^, ^35^ All 33 items on the full version were rated on a 5‐point Likert scale, ranging from “never” (1) to “very often” (5), and the age‐adapted 20‐item version included a reduced 3‐point response scale: “no” (1), “sometimes” (3) and “yes” (5). Mean scores were calculated for each subscale. Both versions were merged for analysis by converting responses on the 20‐item version as follows: 1 = 1, 2 = 3 and 3 = 5. The questionnaires were self‐reported and completed pre‐ and posttreatment in both groups. Both the full 33‐item and the reduced 20‐item DEBQ versions have been used increasingly in paediatric obesity research and shown to have adequate internal consistency, test–retest reliability, factorial validity, and dimensional stability for measuring disordered eating behaviour in children aged 7–17 years.^20^, ^34^, ^35^ In the current study, the Cronbach's *α* coefficient for the 33‐item version was .76 for restrained, .87 for external and .96 for emotional eating at baseline. For the 20‐item version, the Cronbach's *α* coefficient was .79 for restrained, .73 for external and .85 for emotional eating at baseline. These indicate acceptable (>0.7) to excellent (>0.9) internal consistency for the three subscales in the current sample.

### 2.8 Statistical analyses

Data were analysed with IBM SPSS version 26 (IBM Corp.) and Stata version 17 (StataCorp LLC., 2021). Descriptive statistics are expressed as the mean and standard deviation (*SD*) for continuous variables, and as frequency and percentage for categorical variables. Baseline differences between FBSFT and TAU participants, and between children who completed the intervention and those who did not, were tested using *t*‐tests and *χ* ^2^ tests of independence, with the significance level set as 0.05.

Linear mixed models were used to estimate and compare changes from pre‐ to posttreatment under the two treatment conditions. The mixed models included the treatment condition (FBSFT vs. TAU), time (baseline and posttreatment), and a treatment‐by‐time interaction, and were fitted with an unstructured residual covariance structure for all primary and secondary outcomes. Models were checked for heteroscedasticity and normality of residuals. Differences in means and 95% confidence intervals were computed from the fixed effect model parameter estimates. This analytical approach deviates from the original protocol,^19^ but our main goal was to determine whether the change in scores over time differs according to the treatment condition, and this is more straightforward to test and interpret with a treatment‐by‐time interaction within a mixed models framework.^36^ This decision was taken prior to the analysis.

Following the principle of intention to treat, all participants were included in the analyses, irrespective of missing data at any measurement points. Mixed models are not based on balanced data assumption and use all available data on each participant, thus accounting for missing data on a response variable. Under the ‘missing at random’ (MAR) assumption, these models provide unbiased estimates.^37^ Intervention (within‐group) effect sizes were estimated on complete data using Glass's Δ, with pretreatment *SD* as denominator. An effect size is commonly interpreted as small (0.2), moderate (0.5) and large (0.8).^38^

#### 2.8.1 Sample size and statistical power

Power analysis was performed prior to the FABO trial based on two treatment groups (FBSFT and TAU) and three measurement points (pretreatment, and 6‐ and 12‐month posttreatment). For an *α* level of .05, a power of 80%, and a correlation of .5 between measurement points, a sample size of 28–164 subjects would allow to detect moderate (Cohen's *f* = 0.25) to small (Cohen's *f* = 0.10)^38^effects of treatment on the primary outcome over time. Based on number of referrals to the obesity clinic, a total of 120 participants were estimated as a realistic sample size to recruit during the study period, and large enough to detect small to moderate differences in the primary outcome between groups. The sample sizes were calculated with G*Power, version 3.1.3.^39^

#### 2.8.2 Missing data

Anthropometric data at baseline were available from all participants, including dropouts. Anthropometric data posttreatment were available for all completers. Approximately 90% of participants provided accelerometer and questionnaire data at baseline, whilst the percent decreased to 68% posttreatment.

## 3 RESULTS

Participants' baseline characteristics in the FBSFT and TAU groups are presented in Tables 1 and 2. There were no significant group differences at baseline in weight, height, BMI‐related metrics, sleep behaviour, physical activity and eating behaviour (*p*  > .05). The percentage of females in FBSFT and TAU groups was 61.0% and 56.4%, respectively (*p* = .618). Twenty‐two participants (19.3%) dropped out of the study: 12 (20.3%) from the FBSFT group and 10 (18.2%) from the TAU group (*p* = .771). There were no significant differences in age, sex, and BMI SDS between ‘treatment completers’ and ‘dropouts’ (*p*  > .05), although the latter group had higher %IOTF‐25 at baseline (150.5% vs. 144.4%, *p* = .045) (Table S1).

**TABLE 1:** Anthropometric characteristics at baseline by treatment group

	FBSFT group			TAU group			
Variables	*N*	Mean ± *SD*	Min–Max	*N*	Mean ± *SD*	Min–Max	*p* Value*
Age (years)	59	12.6 ± 3.3	5.9–17.7	55	12.6 ± 2.8	6.9–17.4	.975
Weight (kg)	59	80.8 ± 28.9	29.4–165.7	55	82.3 ± 22.4	40.6–114.7	.758
Height (cm)	59	157.1 ± 16.7	112.9–186.4	55	159.3 ± 14.3	130.4–183.7	.457
Height SDS^a^	59	0.6 ± 1.2	−2.5 to 4.2	55	0.7 ± 0.8	−1.3‐4.5	.757
BMI (kg/m^2^)	59	31.9 ± 5.4	22.2–50.0	55	31.7 ± 4.3	23.4–38.9	.826
BMI SDS^a^	59	3.0 ± 0.5	2.2–4.9	55	2.9 ± 0.4	2.1–3.8	.761
%IOTF‐25^b^	59	146.2 ± 14.1	124.1–204.3	55	144.9 ± 11.3	121.6–171.6	.598

Abbreviations: BMI, body mass index; FBSFT, family‐based behavioural social facilitation treatment; Max, maximum; Min, minimum; *SD*, standard deviation; SDS, standard deviation score; TAU, treatment as usual; %IOTF‐25, percentage above the International Obesity Task Force cut‐off for overweight.

^a^ Calculated using the Norwegian growth reference.

^b^ Calculated using the International Obesity Task Force criterion for overweight.

* *p* Value obtained by independent *t*‐test.

**TABLE 2:** Behavioural characteristics at baseline by treatment group

	FBSFT group	TAU group	Group difference	
Behavioural outcome	Mean ± *SD*	Mean ± *SD*	Mean	*p* Value
Sleep duration, 7 days' mean	7:39 (0:57)	7:42 (0:45)	−0: 03	.802
Mid‐sleep time, 7 days' mean	3:32 (1:06)	3:49 (1:23)	−0: 17	.255
Sleep onset time, 7 days' mean	23:23 (1:31)	23:36 (1:36)	−0: 13	.480
Wake‐up time, 7 days' mean	7:43 (0:56)	8:02 (1:17)	−0: 19	.142
Percentage time in MVPA	9.87 (5.62)	8.61 (5.20)	1.26	.245
DEBQ scores				
Emotional eating	1.74 (0.90)	1.78 (0.89)	−0.03	.828
External eating	3.15 (0.88)	2.94 (0.92)	0.21	.243
Restrained eating	2.65 (0.86)	2.74 (0.67)	−0.09	.544

*Note*: All sleep outcomes are reported as hours:minutes. Mid‐sleep time is the midpoint between time of sleep onset and wake‐up time. DEBQ scores are rated on a 5‐point scale, ranging from 1 to 5.

Abbreviations: DEBQ, Dutch Eating Behaviour Questionnaire; FBSFT, family‐based behavioural social facilitation treatment; MVPA, moderate‐to‐vigorous physical activity; *SD*, standard deviation; TAU, treatment as usual.

### 3.1 Primary outcome (change in BMI‐related metrics)

The treatment‐by‐time interaction indicated statistically significant differences in changes from baseline to posttreatment in both BMI SDS (0.19 units, *p* < .001) and %IOTF‐25 (5.48%, *p* < .001) between FBSFT and TAU (Table 3). Furthermore, BMI SDS and %IOTF‐25 decreased significantly in the FBSFT group from baseline to posttreatment (0.16 units, *p* < .001 and 6.53%, *p* < .001), whilst changes in the TAU group were not statistically significant for BMI SDS or %IOTF‐25 (0.03 units, *p =* .30 and −1.04%, *p* = .29) (Table 3).

**TABLE 3:** Changes in outcome variables by treatment group and difference in outcome among the treatment groups from baseline to posttreatment

	Treatment group					
	Mean change from baseline to posttreatment (95% CI)			Time	Mean difference between groups (95% CI)	Group × time
Outcome	FBSFT	TAU	All participants	*p*		*p*
BMI SDS	−0.16 (−0.22; −0.10)	0.03 (−0.03; 0.09)	−0.06 (−0.11; −0.02)	.004	0.19 (0.10; 0.28)	<.001
%IOTF‐25	−6.53 (−8.45; −4.60)	−1.04 (−2.99; 0.90)	−3.79 (−5.16; −2.42)	<.001	5.48 (2.74; 8.22)	<.001
Sleep duration	1.53 (−12.2; 15.26)	−17.95 (−33.73; −2.17)	−8.21 (−18.67; 2.25)	.124	−19.48 (−40.40; 1.44)	.068
Mid‐sleep time	15.43 (−0.55; 31.415)	−10.90 (−29.73; 7.93)	2.27 (−10.08; 14.62)	.719	−26.33 (−51.03; −1.63)	.037
%MVPA	−1.32 (−2.43; −0.21)	−0.98 (−2.28; 0.32)	−1.15 (−2.01; −0.30)	.008	0.34 (−1.37; 2.05)	.696
Restrained eating	0.21 (−0.07; 0.50)	−0.12 (−0.44; 0.20)	0.05 (−0.17; 0.26)	.660	−0.33 (−0.76; 0.10)	.131
External eating	−0.13 (−0.33; 0.07)	−0.09 (−0.31; 0.14)	−0.11 (−0.26; 0.04)	.158	0.04 (−0.26; 0.34)	.782
Emotional eating	0.01 (−0.24; 0.26)	−0.04 (−0.32; 0.24)	−0.01 (−0.20; 0.17)	.876	−0.05 (−0.43; 0.32)	.776

*Note*: All sleep outcomes are reported in minutes. Mid‐sleep time is the midpoint between time of sleep onset and wake‐up time. Restrained eating, external eating, emotional eating are the three subscales of the Dutch Eating Behaviour Questionnaire.

Abbreviations: BMI SDS, BMI standard deviation score; FBSFT, family‐based behavioural social facilitation treatment; TAU, treatment as usual; 95% CI, 95% confidence interval; %IOTF‐25, percentage above the IOTF cut‐off for overweight; %MVPA, percentage of time spent on moderate‐to‐vigorous physical activity.

**FIGURE 2:**
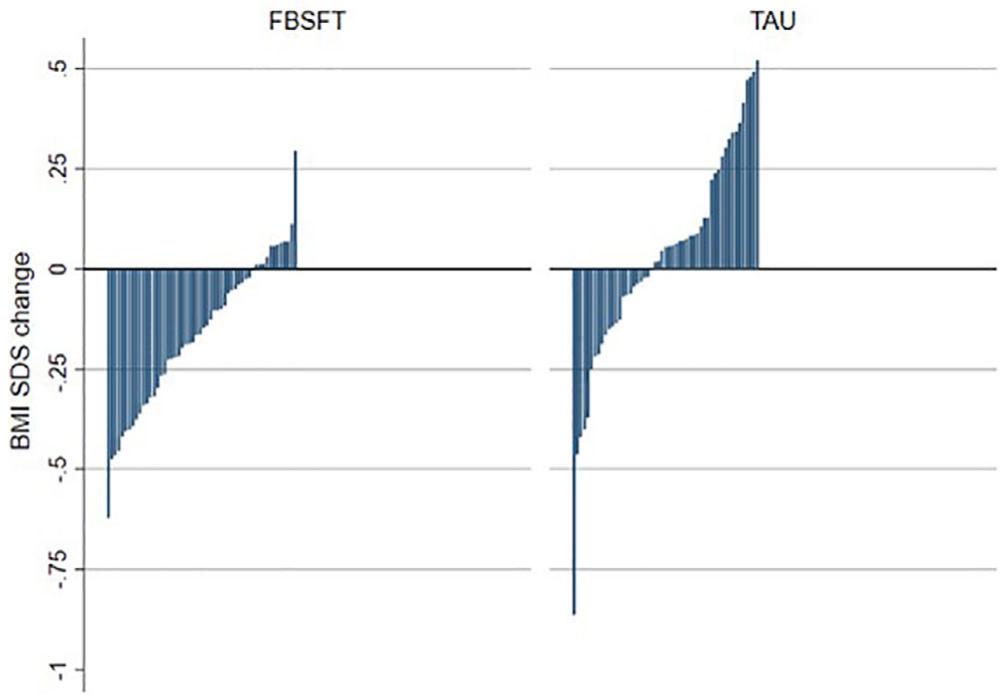
Individual variation in BMI SDS change from pretreatment to posttreatment for family‐based behavioural social facilitation treatment (FBSFT) and treatment as usual (TAU) groups. Each bar represents the change in a single patient

Individual changes in BMI SDS of all participants in both groups are shown in Figure 2. A clinically meaningful reduction in BMI SDS of ≥0.25 was observed in 31.5% (*n* = 17) of the participants in FBSFT and 13% (*n* = 7) in TAU, a significant difference (*χ* ^2^(1) = 5.357, *p* = .021). Intervention effects are presented in Table 4.

**TABLE 4:** Intervention (within‐group) effect sizes

	Within‐group effect size^a^
Variables	Glass Δ
BMI SDS^b^	
FBSFT (*n* = 59)	0.30
TAU (*n* = 55)	0.03
%IOTF‐25^c^	
FBSFT (*n* = 59)	0.46
TAU (*n* = 55)	0.16
Sleep duration, 7 days mean	
FBSFT (*n* = 59)	0.04
TAU (*n* = 55)	0.32
Mid‐sleep time, 7 days mean	
FBSFT (*n* = 59)	0.24
TAU (*n* = 55)	0.09
Emotional eating	
FBSFT (*n* = 59)	0.05
TAU (*n* = 55)	0.10
External eating	
FBSFT (*n* = 59)	0.15
TAU (*n* = 55)	0.05
Restrained eating	
FBSFT (*n* = 59)	0.16
TAU (*n* = 55)	0.25
Percent time in MVPA	
FBSFT (*n* = 59)	0.25
TAU (*n* = 55)	0.14

*Note*: Mid‐sleep time is the midpoint between time for sleep onset and wake‐up time.

Abbreviations: BMI, body mass index; FBSFT, family‐based behavioural social facilitation treatment; MVPA, moderate‐to‐vigorous physical activity; SDS, standard deviation score; TAU, treatment as usual; %IOTF‐25, percentage above the International Obesity Task Force cut‐off for overweight.

^a^ Glass's Δ was calculated by dividing the mean of the difference scores by the pretreatment standard deviation.

^b^ Calculated using the Norwegian growth reference.

^c^ Calculated using the International Obesity Task Force criterion for overweight.

### 3.2 Secondary outcomes

There was a significant difference in changes in sleep timing (operationalized as mid‐sleep time) from pre‐ to posttreatment (−26.3 min, *p* = .037) between the FBSFT and TAU groups (Table 3). The mid‐sleep time increased for FBSFT and decreased for TAU from pre‐ to posttreatment, but neither was statistically significant by itself (FBSFT: *p* = .058, TAU: *p* = .257). For the percentage of time spent in MVPA we observed an overall significant reduction from pre‐ to posttreatment (*p =* .008), but no differences between treatment groups.

There were no significant differences between or within groups for any of the other secondary outcome measures.

## 4 DISCUSSION

This RCT demonstrated that in a public healthcare setting, children with severe obesity receiving FBSFT reduced their BMI SDS and %IOTF‐25 significantly more during the treatment period than children enrolled in TAU. In addition, a larger proportion of FBSFT participants showed a significant reduction in BMI SDS of ≥0.25 from pre‐ to posttreatment. Changes in eating, sleep and physical activity behaviour were minimal, with only changes in sleep timing showing a significant difference between the two groups.

The between group difference in change in BMI SDS (0.19 units) is of similar magnitude as the findings from two recent Cochrane reviews on diet, physical activity and behavioural interventions.^5^, ^7^ These reviews reported a significant pooled treatment effect in favour of the interventions compared to control conditions of −0.06 BMI SDS units in 6–11‐year‐old children^7^ and of −0.13 units in 12–17‐year‐old adolescents.^5^ Interestingly, our study produces better results than majority of studies on behaviour‐based interventions with similar contact hours^8^ and follow‐up period.^5^

Narrowing the comparison to studies on standardized FBT, the pooled result of eight pioneer studies from Epstein et al. shows a BMI SDS change in FBT of −1.20 units at 6 months,^17^ which is considerably larger than in our study. However, all eight studies were efficacy studies conducted in research clinics.^17^ In contrast to these tightly controlled settings our study aimed to assess the response to FBSFT in a regular outpatient clinic where lower treatment effectiveness was expected. The few RCTs on FBT carried out in effectiveness studies up to date have not been able to reproduce the effects reported by Epstein's group.^40^, ^41^ Furthermore, the high mean BMI SDS score at baseline may have influenced the effectiveness. A recent study on FBT compared the BMI SDS change after 4 months of treatment for children with severe obesity and nonsevere obesity.^42^ Children with severe obesity had a mean reduction of −0.20 units, a result aligning with ours. For children with less severe obesity the reduction were of −0.37 units.^42^ Other reasons for the more modest BMI SDS reduction observed in our sample can be related to the experience level of the treatment staff and the modification from mixed (group + individual) format to an individual family format.^15^

In the present study, significantly more participants in the FBSFT group achieved a BMI SDS reduction of ≥0.25 (31.5% in FBSFT group compared to 13% in TAU group). Individual treatment response is an important outcome measure, in addition to mean changes.^43^, ^44^ A previous study found that half of children improved their anthropometric status, despite no mean group change in BMI SDS.^44^ Currently, there is no consensus on thresholds that indicate clinically meaningful changes in BMI SDS among children and adolescents. Suggested reductions in BMI SDS required to improve metabolic health range from 0.1 to 0.5.^26^, ^27^, ^45^ In general, it appears that a reduction in BMI SDS of ≥0.25 is required for clinical effectiveness,^26^, ^43^, ^44^ and larger benefits can be expected with reductions of ≥0.50.^44^ However, another Norwegian study found that even small or modest BMI SDS reductions from ≥0.00 to <0.10 were associated with an improvement in several cardiovascular risk factors.^45^ Therefore, it is likely that any BMI SDS reduction among children with obesity is clinically beneficial,^26^, ^44^ especially in those with severe obesity or obesity‐related comorbidities. Furthermore, it is plausible that children with obesity not receiving treatment will increase in percentage of overweight.^16^

In this study, we presented BMI outcomes in terms of %IOTF‐25, in addition to BMI SDS. Changes in adiposity in children with severe obesity might be difficult to detect using BMI SDS, because large BMI differences corresponds to only small BMI SDS changes.^25^ Therefore, BMI expressed as a percentage of the limits of obesity has been proposed as an alternative measure to BMI SDS, more specifically the %IOTF‐25.^25^ Since the use of %IOTF‐25 as an alternative measure to BMI SDS has recently been suggested,^25^ no directly comparable studies are available. However, a recent US study including 7–11‐year old children,^42^ reported that when using a similar parameter, percent of the 95th percentile of the Centers of Disease Control and Prevention BMI‐reference, the degree of change was found similar irrespective of weight status being overweight/obesity/severe obesity, whereas the reduction in BMI SDS was found lower in the group with severe obesity.^42^

The overall positive results in BMI outcomes with FBSFT versus TAU might be related to treatment content. In contrast to TAU, FBSFT is a structured cognitive behavioural approach that targets both children and parents.^19^ In this study, FBSFT was delivered across an intensive treatment phase with 17 fortnightly sessions, whilst TAU consisted of monthly counselling sessions with a local healthcare nurse and quarterly sessions at the obesity clinic for 1 year. It is possible that a shorter between‐session interval proved advantageous to FBSFT participants, but less so with longer intervals in TAU, suggesting a more concentrated intervention delivery schedule may be beneficial. Notably, the differences exhibited in BMI‐related outcomes between the two groups are most likely driven by their distinct treatment content and targets, indicating the importance of FBSFT and its family‐based approach. Family involvement is a key to treatment success, although its optimal extent remains unclear.^46^ However, parents changing their own behaviours to help their child has been reported as crucial to treatment success,^46^ and this is an important component of FBSFT.

No significant differences for changes in sleep duration, physical activity, or eating behaviour during treatment were observed between the two groups. Analyses of sleep behaviour showed that changes in sleep timing were significantly different between the groups, with a small increase in mid‐sleep time from baseline to posttreatment in the FBSFT group, compared to a small decrease in the TAU group. However, the changes in mid‐sleep time from pre‐ to posttreatment were not statistically significant for either group separately. Therefore, it is unlikely that the observed changes between groups are clinically meaningful. To our knowledge, this study is the first to objectively measure sleep behaviour in school‐aged children receiving FBT for obesity.^23^ Our study was not able to detect meaningful differences in change between groups. However, since obesity and insufficient sleep are bidirectionally associated in children,^22^ we recommend further investigation of sleep as a part of obesity treatment.

Physical activity is addressed similarly in FBSFT and TAU, therefore the nonsignificant between group difference in change was somewhat expected and in line with results from previous studies.^5^, ^7^ However, it is surprising that a significant mean reduction in time spent on MVPA was observed in both groups combined. A wider focus on the barriers that deter children with obesity from engaging in physical activity could possibly strengthen the physical activity component of intervention programmes.^47^

The effects of multidisciplinary treatment for childhood obesity on eating behaviour in children with obesity has until recently been largely unknown.^48^ A systematic review from 2019 concluded that multidisciplinary treatment with a cognitive behavioural component had a positive impact on external and emotional eating, whilst findings for efficacy on dietary restraint were mixed.^48^ Another recent systematic review^49^ reported on five studies using DEBQ emotional eating subscale as a pre‐postmeasure in obesity treatment trials including a dietary component. Two of the studies reported a significant reduction of emotional eating and another study found this change among boys but not among girls. The last two studies reported no change,^49^ as in our study data. In our study, participants in both groups presented with few symptoms of emotional eating at baseline. Therefore, marked favourable changes were not expected. Interestingly, however, healthcare workers involved with the FBSFT group often observed symptoms of emotional eating among participating adolescents, although these behaviours were not reported in questionnaire assessments. One possible explanation for our finding is that awareness and understanding of emotional eating among children and adolescents at baseline might be limited, and self‐report measures may not capture the extent of symptoms of emotional eating experienced. Another explanation is that emotional eating might be considered shameful to report.

Altogether, the results from secondary outcomes (sleep, physical activity and eating behaviour) indicate that observed differences in change in weight outcomes cannot be explained by differences in the lifestyle behaviours measured.

This study has several strengths and limitations. We used an RCT design which is given a high level of evidence for evaluation of treatment options. Because the average BMI SDS at baseline was relatively high, compared to similar RCTs,^1^ with few exclusion criteria, we had the opportunity to investigate the FBSFT approach in a growing patient group of children with the most severe form of obesity.^20^ Dropout rates were comparable in both treatment groups and relatively low (19.3%), compared to rates of 27%–73% previously reported.^50^ Finally, we used objective measures of sleep and physical activity, in contrast to most previous studies that relied on self‐ or parent‐reported data.^5^, ^7^, ^23^

Our study also has some limitations. First, there are no data on energy intake. In the first year of the study, participants were asked to complete a 4‐day food record at baseline and posttreatment, but this was abandoned due to low compliance. It is plausible that the beneficial BMI outcomes in the FBSFT group are due to a reduced calorie intake.^51^ Another limitation is the difference in mean treatment duration between the two groups. Participants in both groups were evaluated as planned at the end of their respective programmes which included a comparable number of sessions, but with the FBSFT group having shorter between‐session intervals than the TAU group. Consequently, the treatment period lasted on average approximately 6 months for the FBSFT group and 12 months for the TAU group. If longer treatment duration is hypothesized to produce better results, we would expect an even larger difference between groups in favour of FBSFT with similar duration of treatment. However, analyses of anthropometric data from the TAU group after 6 months showed the same BMI SDS increase (0.03 units) as after 12 months, and thus did not affect our study conclusions. Finally, we cannot comment on the sustainability of the demonstrated results, and further work on examining long‐term follow‐up data are warranted.

In conclusion, this study demonstrates that among children and adolescents with severe obesity, FBSFT delivered in a public healthcare setting has overall better treatment effects on BMI‐related outcomes, compared to 1 year of TAU. However, changes in the measured lifestyle behaviours were minimal, thus indicating that observed differences in weight outcomes cannot be explained by differences in the included lifestyle behaviours. Considering these findings, expanding access to FBSFT for children and adolescents with severe obesity is an important next step in the treatment of childhood obesity. Alternatively, it may be beneficial to include FBSFT in a stepped approach offered to individuals who do not respond to standard lifestyle treatment.

## CONFLICT OF INTEREST

The authors declare no conflict of interest.

## AUTHOR CONTRIBUTIONS

Hanna F. Skjåkødegård, Yngvild S. Danielsen, Mathieu Roelants, Rachel P. K. Conlon, Denise E. Wilfley and Petur B. Juliusson conceived and designed the study. Hanna F. Skjåkødegård, Yngvild S. Danielsen and Petur B. Juliusson collected the data. Sven J. G. Olsson developed the tailor‐made software for processing physical activity data. Hanna F. Skjåkødegård, Yngvild S. Danielsen, Bente Frisk and Sigurd W. Hystad performed statistical analyses. Hanna F. Skjåkødegård wrote the paper in consultation with Yngvild S. Danielsen, Mathieu Roelants, Bente Frisk, Sigurd W. Hystad, Sven J. G. Olsson, Rachel P. K. Conlon, Denise E. Wilfley and Petur B. Juliusson. All authors discussed the results and contributed to the final manuscript.

## Supporting information


**TABLE S1** Comparison of baseline characteristics between participants who completed the study and dropouts
