# Supplementary material for: Family‐based treatment of children with severe obesity in a public healthcare setting: Results from a randomized controlled trial
Source: Clin Obes. 2022 Feb 25;12(3):e12513. doi: 10.1111/cob.12513 (PMC9286578; doi:10.1111/cob.12513)
Supplement: Supplementary file 1 — TABLE S1 Comparison of baseline characteristics between participants who completed the study and dropouts [file COB-12-0-s001.docx]

**Family-based treatment of children with severe obesity in a public healthcare setting: Results from a randomized controlled trial**

Hanna F. Skjåkødegård^1^ | Rachel P. K. Conlon^2^ | Sigurd W. Hystad^3^ | Mathieu Roelants^4^ | Sven J. G. Olsson^5^ | Bente Frisk^6,7^ | Denise E. Wilfley ^8^ | Yngvild S. Danielsen ^9*^ | Petur B. Juliusson^1,10,11*^

^*^ Yngvild S. Danielsen and Petur B. Juliusson are joint senior authors.

^1^Department of Clinical Science, University of Bergen, Bergen, Norway

^2^Department of Psychiatry, University of Pittsburgh School of Medicine, Pittsburgh, Pennsylvania, USA

^3^Department of Psychosocial Science, University of Bergen, Bergen, Norway

^4^Department of Public Health and Primary Care, KU Leuven, University of Leuven, Leuven, Belgium

^5^Independent researcher, Stockholm, Sweden

^6^Department of Health and Functioning, Western Norway University of Applied Sciences, Bergen, Norway

^7^Department of Physiotherapy, Haukeland University Hospital, Bergen, Norway

^8^Department of Psychiatry, Washington University School of Medicine, St. Louis, Missouri, USA

^9^Department of Clinical Psychology, University of Bergen, Bergen, Norway

^10^Children and Youth Clinic, Haukeland University Hospital, Bergen, Norway

^11^Department of Health Registry Research and Development, Norwegian Institute of Public Health, Bergen, Norway

**Correspondence**

Hanna F. Skjåkødegård, University of Bergen, Department of Clinical Science, N-5021 Bergen, Norway. Email: hanna.skjakodegard.uib.no

**TABLE S1** Comparison of baseline characteristics between participants who completed the study and dropouts

|  | Completed | | Dropouts | |  |
| --- | --- | --- | --- | --- | --- |
| Variables | **N** | **Mean ± SD or %** | **N** | **Mean ± SD or %** | ***P*-value** |
| Age (years) | 92 | 12.5 ± 3.1 | 22 | 12.9 ± 2.9 | 0.632* |
| Gender (% girls) | 92 | 60.9 | 22 | 50.0 | 0.352** |
| BMI SDS^a^ | 92 | 2.9 ± 0.5 | 22 | 3.0 ± 0.5 | 0.621* |
| %IOTF-25^b^ | 92 | 144.4 ± 11.4 | 22 | 150.5 ± 16.8 | 0.045* |

Abbreviations: BMI, body mass index; SD, standard deviation; SDS, standard deviation score; %IOTF-25, percentage above the International Obesity Task Force cut-off for overweight.

^a^Calculated using the Norwegian growth reference.

^b^Calculated using the International Obesity Task Force criterion for overweight.

**P*-value obtained by independent *t*-test indicating differences between treatment groups.

***P*-value obtained by Pearson’s chi-square test.
